# ECPR readiness without perfusionist support: a cross-sectional national survey on circuit preparation, team structure, and training

**DOI:** 10.1186/s12873-026-01766-7

**Published:** 2026-09-11

**Authors:** Eva Mishuris, Alexander Wallraff, Rüdger Kopp, Jan Larmann, Lasse J. Strudthoff, Christian Bleilevens, Patrick Winnersbach

**Affiliations:** 1 Department of Anaesthesiology, Medical Faculty, University Hospital RWTH Aachen, Pauwelsstraße 30, 52074 Aachen, Germany; 2 Department of Heart Surgery, Medical Faculty, University Hospital RWTH Aachen, Pauwelsstraße 30, 52074 Aachen, Germany; 3 Department of Intensive Care, Medical Faculty, University Hospital RWTH Aachen, Pauwelsstraße 30, 52074 Aachen, Germany; 4 Biorheology Research Laboratory, Griffith University, 1 Parklands Dr, Southport, Gold Coast, QLD 4215 Australia

**Keywords:** Extracorporeal cardiopulmonary resuscitation (ECPR), Extracorporeal membrane oxygenation (ECMO), Pre-assembly, Pre-priming, Team structure, Training, Standard operating procedures (SOPs)

## Abstract

**Background:**

Extracorporeal cardiopulmonary resuscitation (ECPR) is a time-critical intervention for selected patients with refractory cardiac arrest. Survival depends on minimizing low-flow duration before ECPR initiation. Timely deployment requires organizational preparedness, trained personnel, and rapid extracorporeal membrane oxygenation (ECMO) circuit readiness. However, data on ECPR organization in centers operating without dedicated perfusionist support are limited.

**Methods:**

A nationwide cross-sectional online survey was conducted among German ECMO centers operating without perfusionist support. The questionnaire assessed circuit readiness, responsible personnel, training frequency, and the availability of standard operating procedures (SOPs).

**Results:**

Responses from 42 centers were evaluated, of which 64% reported performing ECPR (*n* = 27). Most centers (90%, *n* = 38) had low annual ECMO volumes (< 50 cases). ECMO use was largely limited to the Cardiohelp and iLA activve/Xenios Console systems. Setup times were significantly shorter for ECPR than for non-ECPR ECMO: median 15 min (IQR 12–18; range 5–30) versus 30 min (IQR 20–40; range 15–105) (Mann–Whitney U = 46, *p* < 0.0001; Cliff’s delta = 0.77; 95% CI 0.54–0.94). Nurses were more frequently involved in ECMO deployment in ECPR than in non-ECPR settings (59% (16/27 centers) vs. 20% (3/15 centers)). Within ECPR programs, regular training was reported more frequently for nurses than for physicians (87.5% (14/16 centers) vs. 60% (6/10 centers)). Overall, 76% (*n* = 32) of centers reported regular training (at least annually) for responsible personnel, 86% (*n* = 36) had internal SOPs in place, and 69% (*n* = 29) expressed the need for a universally applicable evidence-based SOP. All centers that reported a pre-defined maximum storage duration (*n* = 28) adhered to the Extracorporeal Life Support Organization guidelines regarding maximum storage duration of pre-assembled ECMO systems (30 days).

**Conclusions:**

German ECMO centers without perfusionist support reported substantial heterogeneity in ECPR readiness and operational workflows. ECPR-performing centers were associated with shorter setup times and more frequently reported interdisciplinary team structures and recurrent team training. These findings describe current organizational practices and perceived needs and support the value of interdisciplinary teamwork, structured training, and standardized protocols as organizational components that may facilitate timely, standardized ECPR deployment.

## Background

Cardiac arrest, defined as the sudden cessation of effective cardiac activity, remains a leading cause of mortality worldwide [1]. In case of refractory cardiac arrest (CA), extracorporeal cardiopulmonary resuscitation (ECPR) via veno-arterial extracorporeal membrane oxygenation (VA-ECMO) has emerged as a potential intervention [2, 3]. According to the Extracorporeal Life Support Organization (ELSO) dashboard, more than 38.200 patients have been reported to have received ECPR worldwide as of July 2026 [4], with 50% registered in the last 5 years [5]. Latest randomized clinical trials demonstrated that ECPR can improve neurological outcome and survival [6–8], leading to its inclusion in both national and international resuscitation guidelines for specific patient populations [9].

A critical determinant of survival is the low-flow time, the interval from the onset of cardiopulmonary resuscitation (CPR) to either return of spontaneous circulation (ROSC), the start of ECPR, or death [10]. Evidence suggests that initiating ECPR within 60 min of CA is critical for optimal outcomes [11]. However, ECPR imposes considerable clinical and logistical demands on the ECPR team, which must manage ongoing resuscitation while simultaneously preparing the ECMO circuit and performing cannulation under sterile conditions. This scenario is mentally challenging and extremely time-critical [12].

To optimize the initiation of ECPR support, the ELSO Red Book highlights the utility of wet pre-assembled circuits (corresponds to pre-primed circuits), stating: “A pre-primed circuit is a significant asset in achieving fast on-pump times and should be considered […]” [13]. Maintaining such circuits in a ready-to-use state may substantially reduce low-flow time and thus improve survival and neurological outcomes in patients with refractory CA. Despite these potential benefits, there is currently no universally accepted protocol regarding whether and how ECMO circuits should be pre-assembled for ECPR, nor who holds responsibility for these preparatory steps [14, 15].

Likewise, the composition of an ECPR team is not strictly specified. It is fundamentally multidisciplinary, typically consisting of ECLS specialists (such as perfusionists or ICU nurses) primarily responsible for direct bedside care and ECLS equipment management, alongside ECLS physicians who provide medical expertise, decision-making, and cannulation [13]. The required training and education for these roles are comprehensive, following guidelines informed by organizations like ELSO. A blend of didactic teaching on circuit components, physiology, patient management, and extensive hands-on simulation sessions to practice circuit priming (filling the circuit with fluid), troubleshooting, emergency procedures (e.g., circuit exchange, air entrainment, decannulation), and team communication is typically included [13].

Our research group previously conducted a nationwide survey among German ECMO centers where perfusionists are responsible for ECMO circuit management [16]. This study, as well as the follow-up analysis focusing on pediatric ECMO centers [17], provided important insights into standard practices and training structures in perfusionist-led teams. Building on those findings, the current investigation now shifts focus to ECMO centers that operate without dedicated perfusionist support, aiming to identify the personnel responsible for ECMO circuit preparation, assess their professional qualifications, and evaluate training frequencies as well as the presence and application of standard operating procedures (SOPs).

Moreover, this study allows for comparison of circuit preparation strategies and response times in non-perfusionist-led versus perfusionist-led settings. It also examines the implementation of ECMO mode (ECPR, VA, or VV), commonly used ECMO systems (e.g., Cardiohelp, iLA activve), and circuit pre-assembly approaches. To our knowledge, this is the first survey to systematically investigate ECPR readiness, team composition, and training practices in German centers operating without perfusionist involvement. The findings provide meaningful insights and conclusions on ECPR logistics and ECMO circuit readiness.

## Methods

A cross-sectional anonymous online survey addressing German ECMO centers operating without perfusionists was conducted. The initial identification of ECMO centers and responsible contact persons heading the ECMO facility was based on the online register of the German Interdisciplinary Association for Intensive Care Medicine and Emergency Medicine (DIVI) as of October 24, 2024. All centers listed in the DIVI registry were cross-referenced with the list of centers receiving perfusionist support published on the website of the German Society for Perfusion and Technical Medicine (DGPTM; formerly German Society for Cardiovascular Engineering, DGfK). Centers listed by the DGPTM were excluded from the study. To further ensure that only centers without perfusionist support were included, the resulting list was cross-referenced with the centers approached in our previous study [16]. In addition, the websites of all centers considered for participation were reviewed to confirm the presence of an established ECMO program. A total of 78 centers were invited to participate. Contact information of each targeted department or responsible individual was obtained from the respective center’s website. In cases where contact information was unavailable, a broader search was conducted using Information Retrieval Systems and search engines.

The questionnaire used in this survey was based on a previous survey conducted by our research group, which focused on the pre-assembly practices of perfusionists in German ECMO centers [16], and was adapted to the objectives of this study.

The survey comprised a total of 34 questions, including 13 open-ended and 21 multiple-choice questions. Of the multiple-choice questions, nine allowed unrestricted multiple selections. Overall, 29 of the questions were mandatory. However, owing to the survey design, not all items were presented to every participating center. Therefore, the number of questions answered varied between centers. The complete questionnaire can be found in the supplementary material. The survey was conducted from November 27, 2024, to January 31, 2025, using the online survey platform Typeform [18]. Invitations were distributed via e-mail directly to all participants or their secretaries or through messages on LinkedIn. The invitation e-mail outlined details regarding the study’s purpose, the investigators, the estimated completion time, and data management procedures, adhering to the CHERRIES criteria for online surveys [19]. Completion of the survey was regarded as implicit informed consent for the subsequent analysis of anonymized data. Only complete questionnaires that met the inclusion criteria were included in the analysis.

Statistical analyses and graphical representations of the results were performed using GraphPad Prism software (version 10.4.1). Statistical significance was defined as *p* < 0.05 and determined using the Mann-Whitney U test and the Fisher’s exact test. Cliff’s delta and its respective 95% confidence interval (CI) were calculated using the MetricGate calculator [20]. Categorical variables are reported as counts and percentages. Continuous variables are presented as mean ± standard deviation (SD) to facilitate direct comparison with our previous study by Winnersbach et al. [16], which used the same reporting format. For variables with statistically significant group differences, median, interquartile range (IQR), and full range were additionally reported to reflect the non-parametric distribution of the data.

Declaration of AI and AI-assisted technologies in the writing process.

During the preparation of this work, the authors used ChatGPT to review syntax and orthography to improve readability and language. After using this tool/service, the authors reviewed and edited the content as needed and take full responsibility for the content of the publication.

## Results

Out of 78 contacted centers, 44 replied (56%). Two centers were excluded due to not having met the inclusion criteria. The responses of 42 centers were evaluated (54%).

**Fig. 1:**
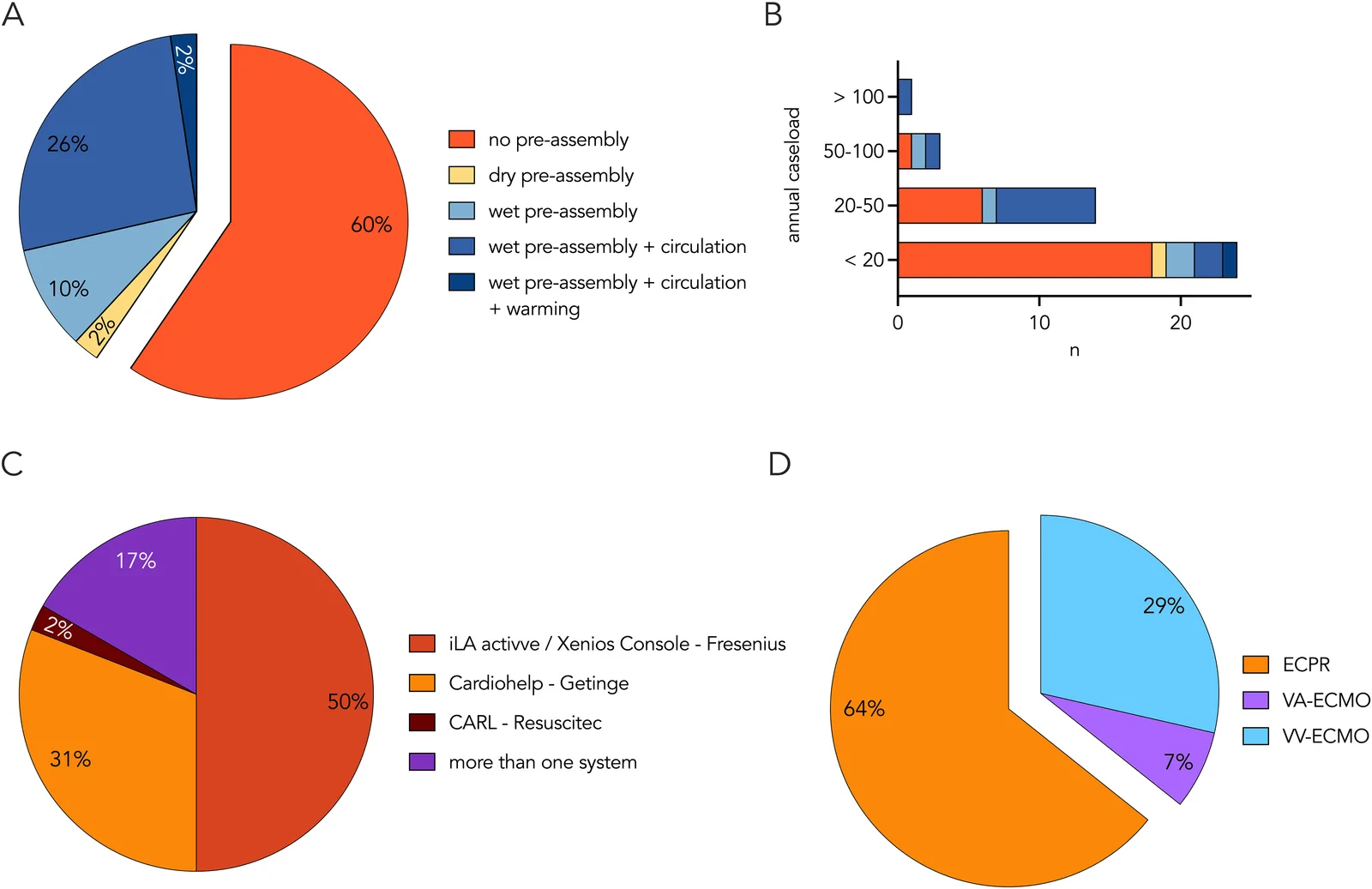
**A**: Degree of ECMO system pre-assembly, percentages of a total of *n* = 42. **B**: Degree of ECMO system pre-assembly depending on the annual caseload. **C**: ECMO systems used. **D**: Application mode of ECMO, categorized according to the most complex configuration applied

Figure 1A shows the degree of pre-assembly in the responding centers. 60% (*n* = 25) do not pre-assemble ECMO systems in advance, whereas 40% (*n* = 17) hold pre-assembled ECMO systems readily available to various extents: dry pre-assembly (2%, *n* = 1), wet pre-assembly (10%, *n* = 4), wet pre-assembly with circulation (26%, *n* = 11), and wet pre-assembly with circulation and warming (2%, *n* = 1).

Figure 1B displays the degree of pre-assembly in relation to the annual caseload. Overall, 57% (*n* = 24) of participating centers reported an annual caseload of less than 20. The majority of centers with a caseload of less than 20 per year does not pre-assemble ECMO systems (75%), while the proportion of centers with wet pre-assembled ECMO systems increases with the annual caseload.

Figure 1C depicts the utilized ECMO systems. 50% (*n* = 21) of the participating centers use iLA activve/Xenios Console (Fresenius), whereas 31% (*n* = 13) use Cardiohelp (Getinge). Other responses included the use of more than one system (17%, *n* = 7) or CARL (Resuscitec; 2%, *n* = 1).

Figure 1D illustrates the ECMO configurations used in the responding centers, categorized according to the most complex configuration applied. A majority of centers (64%, *n* = 27) reported performing ECPR. For non-ECPR ECMO, veno-arterial configuration is applied in 7% (*n* = 3) of responding centers, while veno-venous ECMO alone is performed by 29% (*n* = 12).

**Fig. 2:**
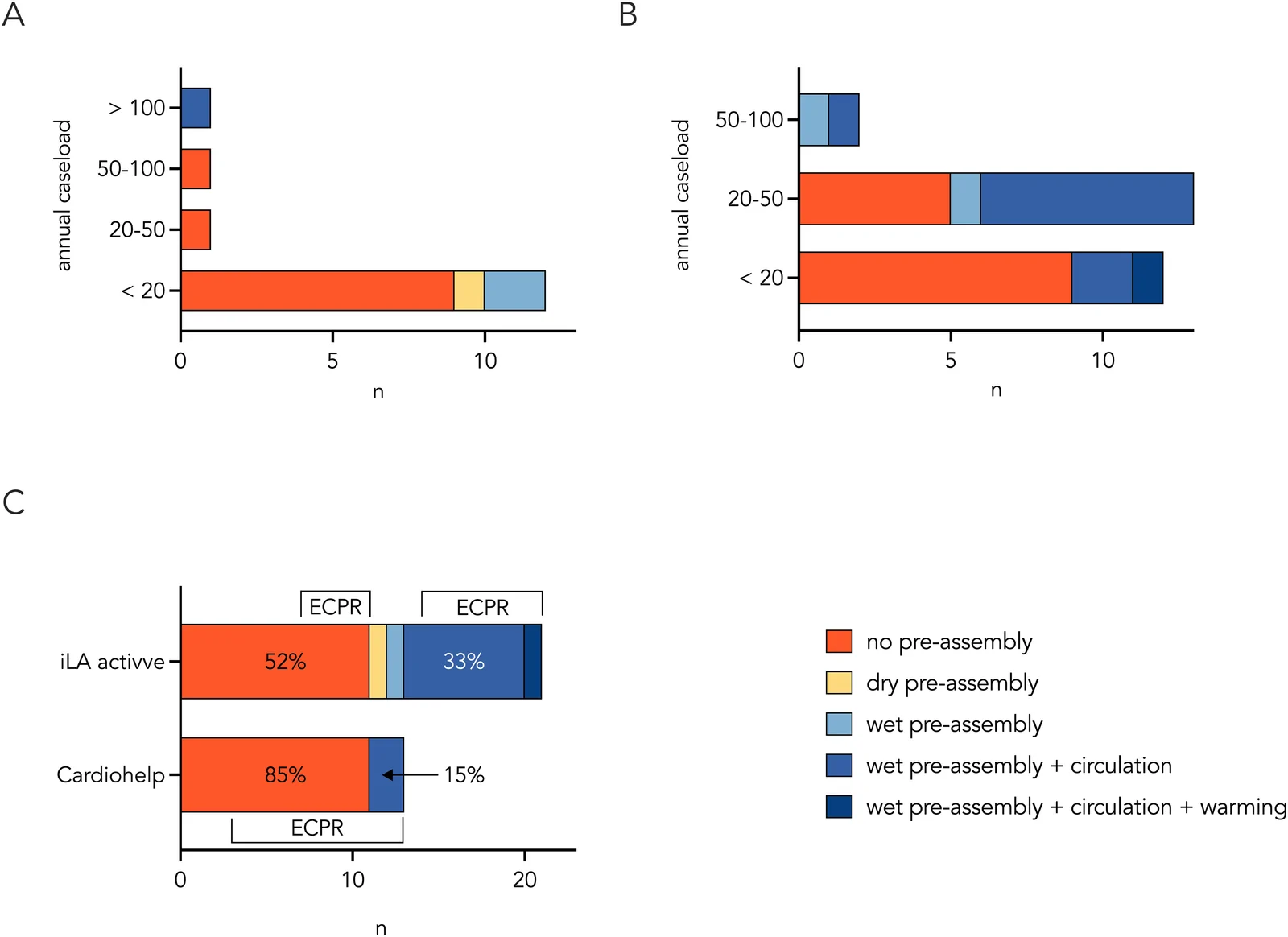
Degree of ECMO system pre-assembly stratified by center volume and the employed systems. **A**: Non-ECPR ECMO (VV- and VA-ECMO, *n* = 15). **B**: Extracorporeal cardiopulmonary resuscitation (ECPR, *n* = 27). **C**: Distribution of Cardiohelp and iLA activve according to the degree of assembly and the usage for ECPR

Figure 2 presents the degree of ECMO system pre-assembly stratified by the annual caseload (Fig. 2A and B) as well as the system used, dependent on the degree of ECMO system pre-assembly (Fig. 2C).

Figure 2A illustrates data from ECMO centers performing non-ECPR ECMO (VV- and VA-ECMO, *n* = 15). A vast majority of 73% (*n* = 11) reported not holding an ECMO system readily available (no pre-assembly).

Figure 2B displays data related to centers performing ECPR (*n* = 27). In the subgroup of centers with fewer than 20 cases per year (*n* = 12), 75% (*n* = 9) reported not holding pre-assembled ECMO systems available. Out of the centers with an annual caseload between 20 and 50 patients (*n* = 13), 38% (*n* = 5) do not pre-assemble ECMO systems.

Figure 2C presents the distribution of Cardiohelp and iLA activve systems, utilized by the majority of centers (cf. Figure 1C), according to the degree of ECMO pre-assembly. The brackets mark the ECPR application for the respective ECMO system.

Most centers that use Cardiohelp do not pre-assemble the ECMO system (85%, *n* = 11). Wet pre-assembly and circulation are applied in the other cases (15%, *n* = 2). The vast majority of centers that employ the Cardiohelp system perform ECPR, as shown by the bracket (77%, *n* = 10). Overall, 52% (*n* = 11) of centers that use the iLA activve system do not pre-assemble in advance, whereas 33% (*n* = 6) utilize wet pre-assembly and circulation. In about half the centers (52%, *n* = 11), ECPR is performed as indicated by the brackets. For all non-ECPR applications (VV- and VA-ECMO, *n* = 13), the iLA activve system was more frequently used (77%, *n* = 10).

**Fig. 3:**
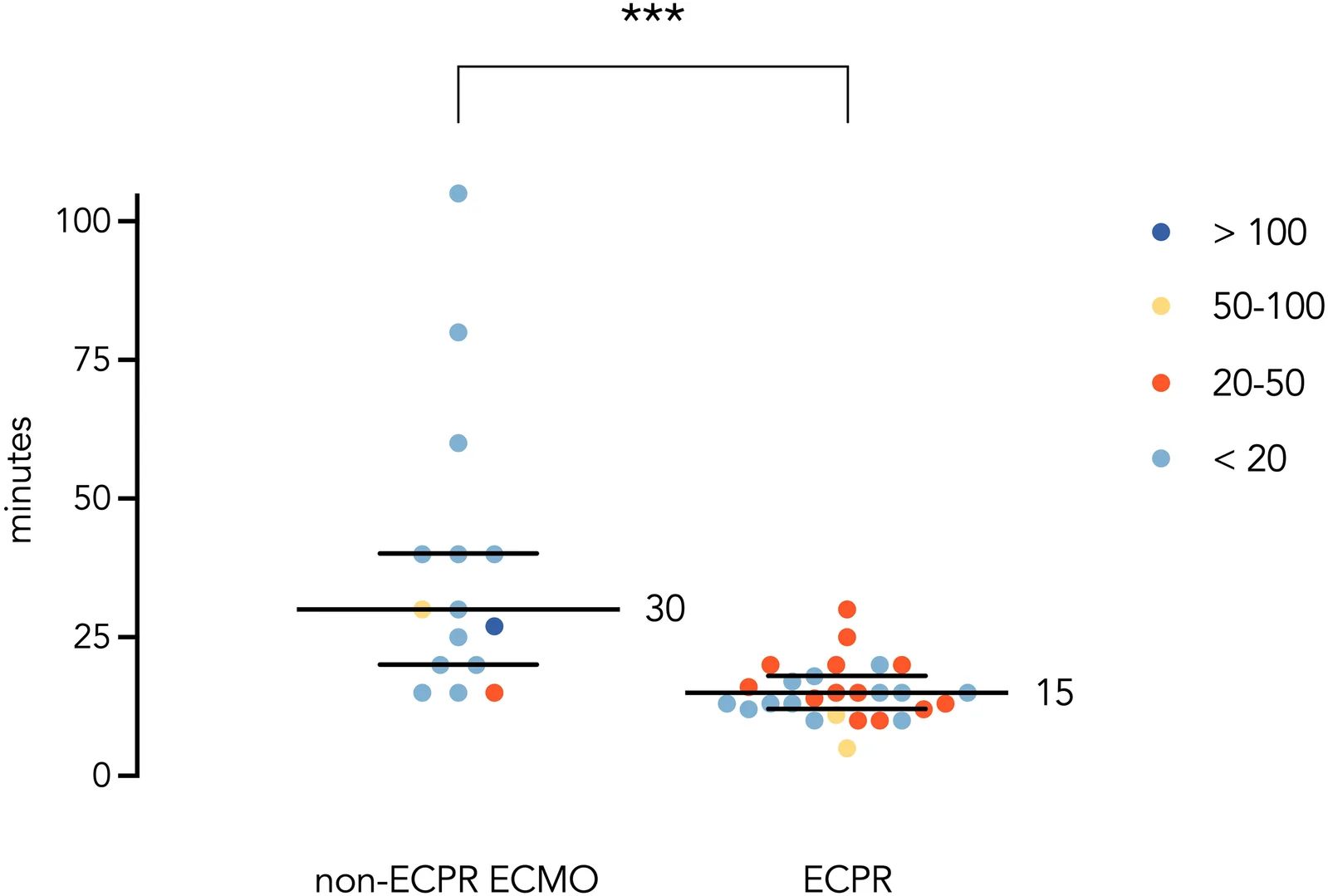
ECMO setup times, grouped by center volume. Left: setup time in centers that use non-ECPR ECMO (*n* = 15). Right: setup time in centers that use ECPR (*n* = 27). Data is reported as median with interquartile range. *** *p* < 0.0001

Figure 3 illustrates the ECMO system setup time in reference to center volume. Setup time was defined as the combined time required for circuit assembly and priming and was calculated separately for each responding center. Setup times were significantly shorter for ECPR (*n* = 27) compared with non-ECPR ECMO (*n* = 15) (U = 46, *p* < 0.0001). The median reported setup time was 15 min (IQR 12–18; range 5–30) for ECPR and 30 min (IQR 20–40; range 15–105) for non-ECPR ECMO (Cliff’s delta = 0.77; 95% CI 0.54–0.94). The mean setup time was 15 ± 5 min for ECPR compared with 37.5 ± 26 min for non-ECPR- ECMO.

**Fig. 4:**
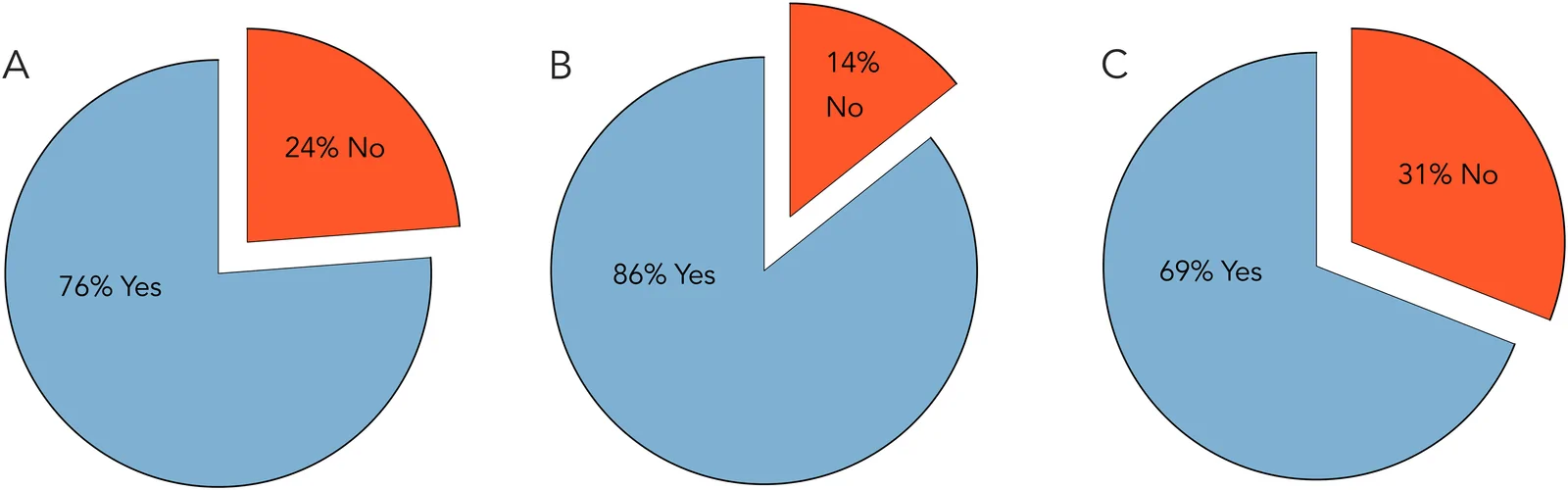
Training and standard operating procedure (SOP). **A**: Training for ECMO setup. **B**: Internal SOP in place. **C**: Need for a universally applicable and evidence-based SOP

Figure 4 summarizes findings regardless of ECMO configuration. The responses of the centers applying ECPR versus VV- and VA-ECMO were proportional without significant differences.

Figure 4A presents the percentages of centers that offer training for setting up the ECMO systems. Overall, 76% (*n* = 32) of responding centers in this study reported offering regular training for responsible personnel.

Figure 4B shows the proportion of centers that have an internal standard operating procedure (SOP) for ECMO system assembly (Fig. 4B) in place, which was the case in 86% (*n* = 36) of all centers.

Figure 4C demonstrates the need for a universally applicable and evidence-based SOP, which was considered useful by 69% (*n* = 29) of all centers

**Fig. 5:**
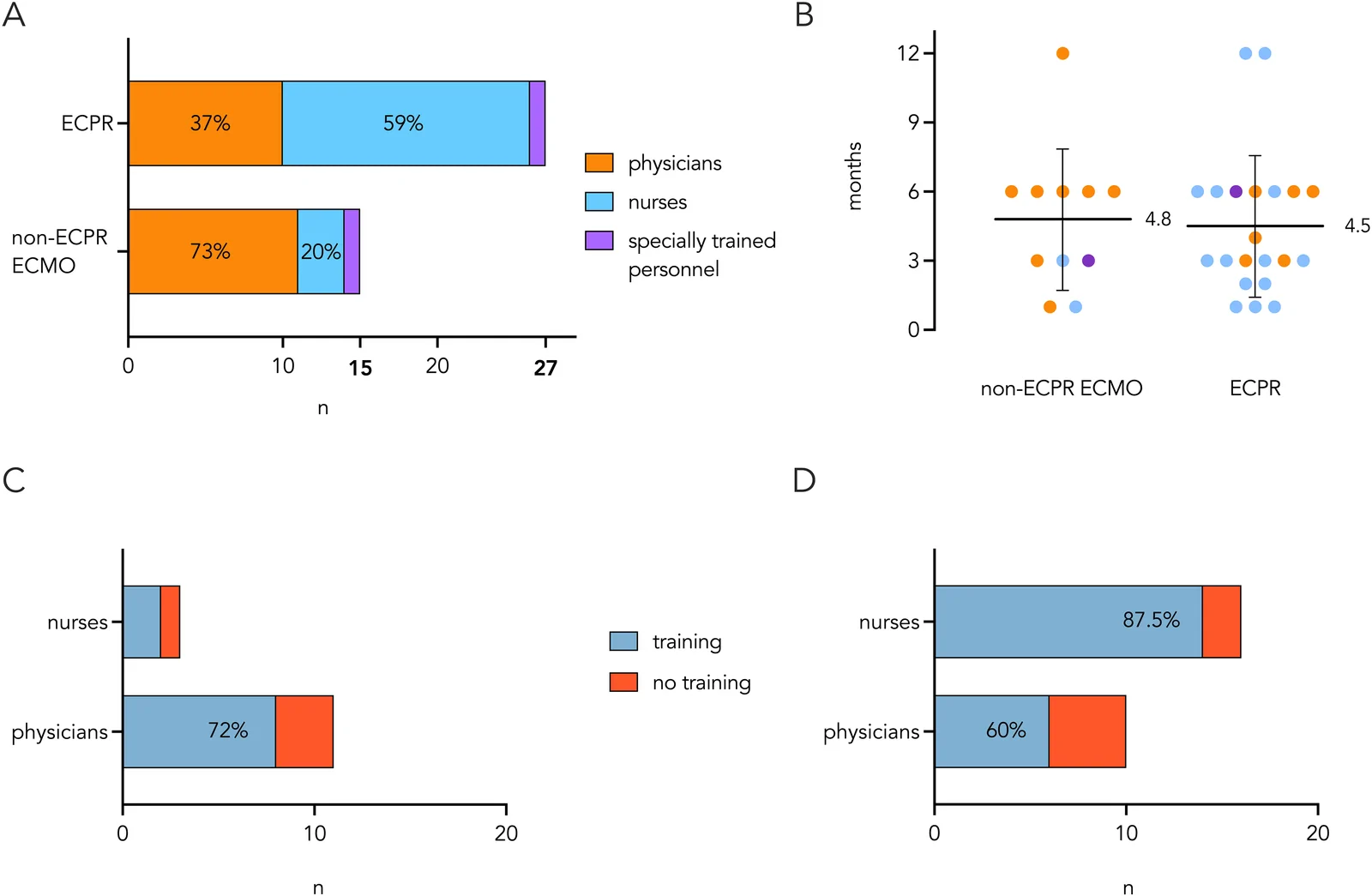
Responsible personnel and training. **A**: Responsible personnel for ECMO setup. **B**: Training intervals. Data is reported as mean ± standard deviation. **C**: Training of responsible personnel for non-ECPR applications. **D**: Training of responsible personnel for ECPR

Figure 5A illustrates the personnel involved in the setup of the ECMO system per ECMO configuration. For non-ECPR ECMO, physicians are in charge of ECMO setup in 73% (*n* = 11) of the centers, whereas this portion is smaller for ECPR (37%, *n* = 10). Nurses manage the ECMO system setup for ECPR in 59% (*n* = 16) of the centers. The subgroup of specially trained personnel included physician assistants (PAs) and medical assistants.

Figure 5B displays the training intervals for the responsible staff for the respective ECMO configuration. Those centers which provide training have implemented training at least once a year (*n* = 32). The mean training interval for non-ECPR ECMO was 4.8 ± 3.1 months across the institutions and 4.5 ± 3.1 months for ECPR.

Figure 5C and D compare whether personnel responsible for ECMO assembly undergo regular training, focusing on differences between nurses and physicians. Figure 5C presents the data for non-ECPR applications (VV- and VA-ECMO). Regular training was reported in 72% (*n* = 8) of centers with physicians being responsible, and in 66.6% (*n* = 2) when nurses were in charge. Figure 5D shows the corresponding data for ECPR: 87.5% (*n* = 14) of nurses and 60% (*n* = 6) of physicians reported receiving regular training.

**Fig. 6:**
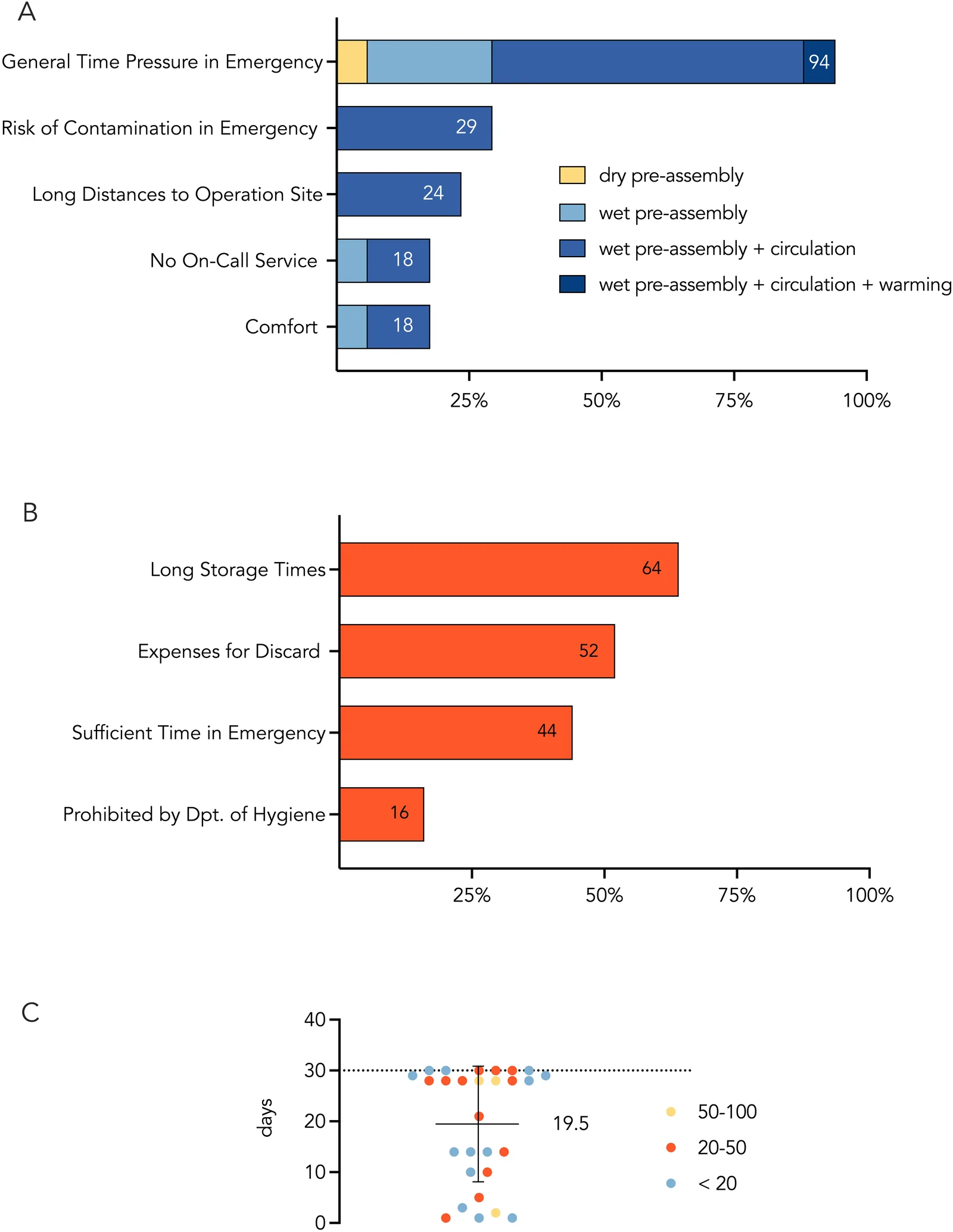
**A**: Reasons for system pre-assembly. **B**: Reasons against system pre-assembly. **C**: Maximum storage duration for wet pre-assembled ECMO systems in days. Data is reported as mean ± standard deviation

Figure 6A shows the most frequently reported reasons for maintaining pre-assembled ECMO systems. These include general time pressure in emergencies (94%), risk of contamination during emergency procedures (29%), long distances to the operation site (24%), absence of an on-call service (18%), and overall convenience (18%). Only centers that hold ECMO systems readily available were able to provide reasons for doing so (*n* = 17).

Figure 6B illustrates reasons for not holding ECMO systems readily available (*n* = 25). Reasons include long storage durations (64%), high disposal costs (52%), sufficient time availability during emergencies (44%), and restrictions imposed by the department of hygiene (16%).

Figure 6C shows the maximum storage duration of wet pre-assembled ECMO systems. This question was answered by centers that either routinely maintained wet pre-assembled systems to various extents or stored circuits after assembly and priming when they had not been used during an emergency (*n* = 31). Of these centers, 28 (90%) reported a pre-defined maximum storage duration. The reported maximum did not exceed 30 days, with a mean storage duration of 19.5 ± 11.3 days.

## Discussion

This national survey provides an overview of how German ECMO centers without perfusionist support organize circuit readiness, team responsibilities, and training for ECPR and other ECMO applications. Three findings are particularly relevant: first, ECPR is established in a substantial proportion of centers (64%); second, circuit readiness strategies vary considerably, particularly concerning pre-assembly; and third, ECPR appears to be associated with greater nursing involvement and shorter setup times than non-ECPR ECMO applications.

The proportion of centers performing ECPR (64%) underscores that ECPR is not confined to highly specialized units, but is part of routine practice in a relevant subset of ECMO centers without perfusionists. At the same time, most participating centers (90%) reported comparatively low annual ECMO case volumes (< 50 cases per year). This contrasts with our earlier survey [16] of perfusionist-supported centers (45% < 50 cases per year) and likely reflects structural differences between hospital types. Rather than interpreting this as lower expertise, it may indicate that lower-volume centers provide important regional access to advanced extracorporeal support [21]. For ECPR in particular, this is clinically relevant, as timely access may outweigh referral to a distant high-volume center when low-flow time is the dominant determinant of outcome [10, 11].

A central finding is the marked heterogeneity in pre-assembly practices. More than half of ECPR-performing centers (52%) did not maintain pre-assembled ECMO systems. While pre-assembled circuits theoretically reduce deployment time in CA, they also require balancing potential time savings against concerns regarding sterility during storage, the risk of contamination, possible component degradation over time, compliance with local infection-control policies, and the economic implications of discarding unused circuits [22, 23]. Consistent with these considerations, the survey indicates that decisions against pre-assembly are largely driven by pragmatic considerations, including storage duration, hygiene concerns, and disposal costs (cf. Figure 6). Conversely, centers that maintain ready-to-use systems primarily cited general time pressure in emergencies as the determining factor. The absence of a consistent relationship between caseload and pre-assembly strategy further supports the interpretation that these decisions are shaped predominantly by local protocols.

A considerable degree of variability was observed in the reported setup times for ECMO systems. The assembly duration for the ECPR configuration was significantly shorter than that observed in the non-ECPR ECMO cohort. The average setup time for ECPR, which did not appear to be substantially influenced by the type of system used, closely aligned with data from previous studies [12, 16]. From a clinical standpoint, the significantly shorter setup times reported for ECPR compared with non-ECPR ECMO are particularly relevant. Given the time-critical nature of CA, this finding may reflect workflow adaptions to prioritize rapid circuit deployment in ECPR scenarios.

Team structure emerged as another key factor. Circuit setup was most commonly performed by physicians and nurses, with substantially higher nursing involvement in ECPR (59%) compared with non-ECPR ECMO (20%). This likely reflects the urgency and complexity of ECPR scenarios where parallel task execution is crucial [24]. In this context, nursing participation appears to be a functional requirement rather than a substitution for perfusionist expertise. International data suggest that trained nurse ECMO specialists can safely contribute to circuit management and may increase program capacity when perfusionist availability is limited [25–28]. Nevertheless, these models typically remain dependent on perfusionists for training, quality assurance, and complex troubleshooting. The present findings are consistent with an interdisciplinary team model as a central component of non-perfusionist ECPR programs.

Training structures further support this interpretation. A high proportion of centers reported regular training (76%). Among ECPR-performing centers in which nursing staff were responsible for circuit setup (59%), 87.5% reported providing regular training for these nurses. Notably, training rates were higher among nursing staff (87.5%) than among physicians (60%) in the ECPR cohort, which may reflect differences in procedural responsibility and repetition frequency. The co-occurrence of nursing responsibility and reported regular training may indicate that some centers pair task allocation with targeted education. Regardless, the data suggest that structured and recurrent training is a critical prerequisite for maintaining ECPR readiness.

The high prevalence of internal SOPs (86%), combined with a strong perceived need for a national or standardized SOP (69%), further underscores the importance of formalized processes. Compared with perfusionist-supported centers [16], non-perfusionist centers more frequently reported having written SOPs. A possible explanation for this discrepancy is that specialized centers with perfusionist involvement adhere to implicit standards, such as ELSO guidelines, even in the absence of a formal written SOP. At the same time, variability in practice, particularly regarding pre-assembly and storage, indicates that existing guidance is either insufficiently specific or inconsistently implemented. Development of evidence-based, flexible SOP frameworks may therefore improve consistency without limiting necessary local adaptation.

Storage practices highlight this issue. Among centers that reported a pre-defined maximum storage duration, all adhered to the ELSO-recommended maximum storage duration of 30 days for wet pre-assembled systems [29], and most reported more conservative internal limits. In contrast, perfusionist-supported centers occasionally reported longer storage durations [16]. While limited evidence suggests that safe storage for up to six weeks may be feasible [15], the lack of standardized guidance may contribute to variability in clinical practice. The implementation of an evidence-based and universally applicable SOP, aligned with ELSO recommendations, could help ensure consistency and safety in storage practices across centers.

This study has several limitations. First, as an anonymous survey, it is subject to non-reporting and selection bias. The response rate of 54% introduces the possibility of selection bias, as participating centers may have been more organized, better trained, or more engaged in ECPR than non-participating centers, potentially leading to an overestimation of national ECPR readiness. Second, respondent characteristics were not systematically collected. Therefore, and due to the anonymous nature of the survey, duplicate submissions could not be detected or excluded and answers may reflect different levels of technical expertise or institutional oversight. Third, all data were self-reported and were not independently audited or validated. In particular, reported setup times may reflect estimated routine practice, retrospective institutional experience, or best-case assumptions rather than prospectively measured emergency performance. It was not possible to ascertain how respondents interpreted individual survey items or whether local definitions of concepts such as ECPR readiness, assembly time, and priming time were applied consistently across centers. Fourth, centers were classified according to their highest level of ECMO capability. As ECPR centers may also perform VA- and VV-ECMO, comparisons between ECPR and non-ECPR centers may have been influenced by overlapping clinical practice. In addition, differences in center characteristics, like center volume, equipment type, pre-assembly strategy, staffing structure, or local protocols may have contributed to the observed differences. Some subgroup analyses were based on relatively small numbers of centers, which may limit the robustness of these findings. Fifth, the study did not assess patient-level outcomes, including low-flow duration, cannulation success, complications, survival, or neurological outcome. Therefore, the findings should be interpreted as a descriptive analysis of current practice rather than evidence of superiority of specific organizational strategies. Finally, the findings reflect practices in German non-perfusionist ECMO centers and may therfore not be generalizable to other healthcare systems. Further investigations towards the safety and patient outcome of ECPR in non-perfusionist centers are warranted.

Looking ahead, new generations of ECMO systems, such as Xenios 2.0 and Cardiohelp II, offer guided illustrations of step-by-step workflows for priming and other procedural steps, intended to improve usability and decision support [30, 31]. It is yet to be observed how these advances in ECMO systems will influence clinical applications and logistics.

## Conclusions

This national survey provides the first comprehensive overview of current organizational practice and perceived needs related to ECPR readiness in German ECMO centers operating without perfusionist support. ECPR capability was reported by a substantial proportion of institutions (64%). ECPR workflows were associated with significantly shorter self-reported setup times compared with non-ECPR ECMO applications. In addition, centers performing ECPR more frequently reported interdisciplinary team structures, particularly involving regularly trained nursing staff. Centers consistently implemented internal SOPs and expressed a strong demand for evidence-based standards. These findings describe current organizational practices and perceived needs rather than demonstrating improved clinical safety or patient outcomes. They support the value of interdisciplinary teamwork, structured training, and standardized protocols as organizational components that may facilitate timely and standardized ECPR deployment, particularly in settings lacking perfusionist expertise. The development of unified SOP frameworks aligned with ELSO recommendations may represent an important step toward improving consistency in ECMO practice. Future prospective studies should evaluate whether specific readiness strategies are associated with reduced low-flow duration, improved safety, and patient-centered outcomes.

## Data Availability

The datasets used and/or analysed during the current study are available from the corresponding author on reasonable request.

## Abbreviations

CA: Cardiac Arrest
CPR: Cardiopulmonary Resuscitation
DGfK: German Society for Cardiovascular Engineering
DGPTM: German Society for Perfusion and Technical Medicine
DIVI: German Interdisciplinary Association for Intensive Care Medicine and Emergency Medicine
ECMO: Extracorporeal Membrane Oxygenation
ECLS: Extracorporeal Life Support
ECPR: Extracorporeal Cardiopulmonary Resuscitation
ELSO: Extracorporeal Life Support Organization
ICU: Intensive Care Unit
PA: Physician Assistant
ROSC: Return of Spontaneous Circulation
SOP: Standard Operating Procedure
VA-ECMO: Veno-Arterial Extracorporeal Membrane Oxygenation
VV-ECMO: Veno-Venous Extracorporeal Membrane Oxygenation
